# Search for a parity-violating long-range spin-dependent interaction

**DOI:** 10.1073/pnas.2512538122

**Published:** 2025-10-07

**Authors:** Xing Heng, Zitong Xu, Xiaofei Huang, Dinghui Gong, Guoqing Tian, Wei Ji, Jiancheng Fang, Dmitry Budker, Kai Wei

**Affiliations:** ^a^Institute of Large-Scale Scientific Facility, School of Instrumentation Science and Opto-Electronics Engineering, Beihang University, Beijing 100191, China; ^b^The Center for Quantum Technologies, School of Physical and Mathematical Sciences, Nanyang Technological University, Singapore 639798, Singapore; ^c^State Key Laboratory of Nuclear Physics and Technology, School of Physics, Peking University, Beijing 100871, China; ^d^Hefei National Laboratory, Hefei 230088, China; ^e^Institute for Physics, Johannes Gutenberg University, Mainz 55128, Germany; ^f^Helmholtz-Institute, GSI Helmholtzzentrum fur Schwerionenforschung, Mainz 55128, Germany; ^g^Department of Physics, University of California at Berkeley, Berkeley, CA 94720-7300; ^h^Hangzhou Extremely Weak Magnetic Field Major Science and Technology Infrastructure Research Institute, Hangzhou 310051, China

**Keywords:** parity violation, hybrid spin-resonance, atomic comagnetometer, beyond-Standard-Model interactions

## Abstract

High-sensitivity quantum sensors are a promising tool for experimental searches for beyond-Standard-Model interactions. Here, we demonstrate an atomic comagnetometer operating under a resonantly-coupled hybrid spin-resonance (HSR) regime to probe P-odd, T-even interactions. The HSR regime enables robust nuclear-electron spin coupling, enhancing measurement bandwidth and stability without compromising the high sensitivity of spin-exchange relaxation-free magnetometers. To minimize vibration-related noise from velocity-modulated sources, we implement a multistage vibration isolation system, achieving a vibration-related noise reduction exceeding 700-fold. We establish new constraints on vector-boson-mediated parity-violating interactions, improving experimental sensitivity by three orders of magnitude compared to previous limits. The new constraints complement existing astrophysical and laboratory studies of potential extensions to the Standard Model.

## Significance

Parity-violating interactions mediated by exotic bosons could reveal physics beyond the Standard Model. We develop a hybrid spin-resonance (HSR) atomic sensor with 700-fold vibration suppression, achieving three orders-of-magnitude sensitivity gain over prior limits. Our measurements establish the strongest constraints on axial-vector couplings between nucleons (0.03–400 m range), resolving critical gaps in low-energy symmetry tests. By synchronizing nuclear-electron spin dynamics, this work advances quantum sensing for dark matter searches and exotic force detection, bridging atomic parity studies with new physics exploration through precision spin-interaction metrology.

The discovery of parity violation in experiments such as the pioneering study of *β* decay of ^60^Co (1) and atomic parity violation (2, 3) fundamentally reshaped our understanding of particle physics and contributed to the establishment of the Standard Model. This phenomenon provides a unique opportunity to test the Standard Model through low-energy precision measurements. Ongoing research explores parity violation in atoms, such as cesium (4, 5), francium (6), and ytterbium (7, 8). In these systems, parity violation arises from the exchange of Z-bosons—the mediators of “neutral current” weak interactions—which are distinct from the “charged current” interactions mediated by W^±^ bosons responsible for the *β* decays as studied in C. S. Wu’s famous experiment (1). Further exploration of neutral-current parity violation also sheds light on the potential existence of “new” Z’-bosons, as well as Z’-boson-mediated exotic interactions (9).

It is proposed that new exotic spin-dependent forces may exist and that the corresponding interaction potentials may be classified into 16 terms based on their symmetry properties (10). These interactions could be mediated by Z’ or spin-0 particles such as the axion (11); some of them could violate parity. Investigation of such interactions could also illuminate the dark matter problem because both Z’ and axions are promising dark-matter candidates. The exotic forces are then classified according to their physical coupling constants, providing a unified framework for studying the effects of hypothetical bosons and their interactions (12, 13). Among these interactions, certain terms dominate in experimental sensitivity, with the axial-vector and vector couplings exhibiting a velocity-dependent parity-violating component (13):

[1] $$V_{\mathrm{PV}}=\frac{g_{A}g_{V}\hbar }{4\pi }(\hat{\sigma }\cdot v)\frac{e^{-r/\lambda }}{r},$$

where ℏ is the reduced Planck constant, $\hat{\sigma }$ is the Pauli-matrix vector of the sensing fermion, v and r are the relative velocity and distance between the sensing fermion and the source fermion, $\lambda =\hbar /m_{b}c$ is the force range, $m_{b}$ is the mass of Z’, and c is the speed of light. This potential corresponds to *V*_12+13_ in (10, 13), with the coupling-strength constant $g_{A}g_{V}$ related to the coefficient $f_{12+13}$ as ${f_{12+13}}^{ij}=2{g_{A}}^{i}{g_{V}}^{j}\times \left(1+\frac{m_{i}}{m_{j}}\right)$, where *i*, *j* label various fermion pairs (e.g., *e−N*, *n−N*, *p−N*). Current astrophysical observations exhibit gaps in the constraints for spin-dependent interactions, motivating direct tests via tabletop experiments. (13, 14). Parity violation measurements in atoms are effective tools for probing forces down to the nuclear scale (15). Mesoscopic techniques, such as NV centers, can search for such forces at the micrometer scale (16), while atomic magnetometers are sensitive to forces at the centimeter scale (17). At the opposite extreme, experiments that use the Earth or Moon as the source can search for forces with ranges exceeding the kilometer scale and extending up to 10^10^ km (18, 19).

In this work, we utilize two lead blocks with a high mass density as the source and employ a state-of-the-art spin exchange relaxation free (SERF) comagnetometer with an ultrahigh energy resolution (14, 20–26) as the sensor to search for exotic particles and velocity-dependent parity-violating interactions. By operating the SERF comagnetometer in the resonantly-coupled hybrid spin-resonance (HSR) regime, we significantly enhance the measurement bandwidth, leading to improved stability while preserving the high sensitivity of the SERF magnetometer. Vibrations induced by the rotating masses are a noise source for these measurements, which we mitigate with a multistage scheme that combines a vibration isolated foundation with a vacuum system enclosing the sensor. This provides a more than 700-fold suppression of the vibration-related noise. We establish the most stringent experimental constraints over a force range of 0.03 to 400 m, particularly improving upon previous limits (17, 21) by three orders of magnitude at λ=5 m.

## Results

### Experimental Setup.

**Fig. 1.**
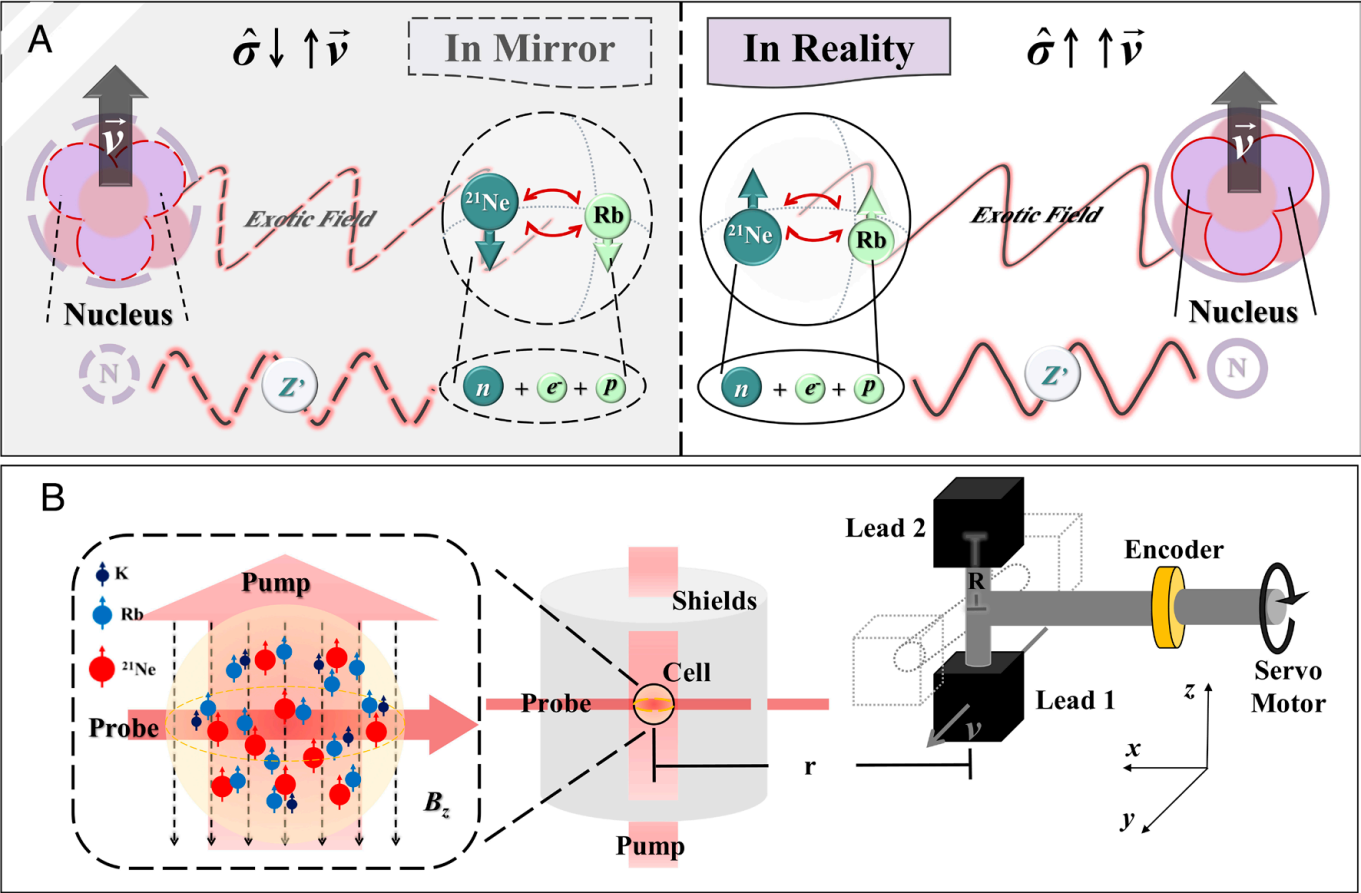
Principle of the experiment. (*A*) Parity can be tested by the exotic spin- and velocity-dependent interaction *V*_12+13_. Notably, the induced pseudomagnetic field exhibits a sign change upon spatial inversion, highlighting the inherent asymmetries explored in this investigation. (*B*) Experimental setup. The servo motor drives two cubic lead blocks at a rotation frequency of 3 Hz, inducing a pseudomagnetic field sensed with an ensemble of polarized hybrid spins. When a lead block reaches the lowest position, the center of the block is in the horizontal plane of the atomic reservoir (vapor cell), at which point the centers of the lead block and the vapor cell are separated by a distance *r* = 52.5 cm.

Parity violation by the exotic force is illustrated in Fig. 1*A*. In the mirror-reflected framework, the spins exhibit inverted polarization compared to the physical reality, while the velocity does not, which leaves them parallel in reality and antiparallel in the mirror. Therefore, the product of velocity and spin changes sign by mirror reflection (and the parity operation), which indicates the parity non-conservation of Eq. **1**.

**Fig. 2.**
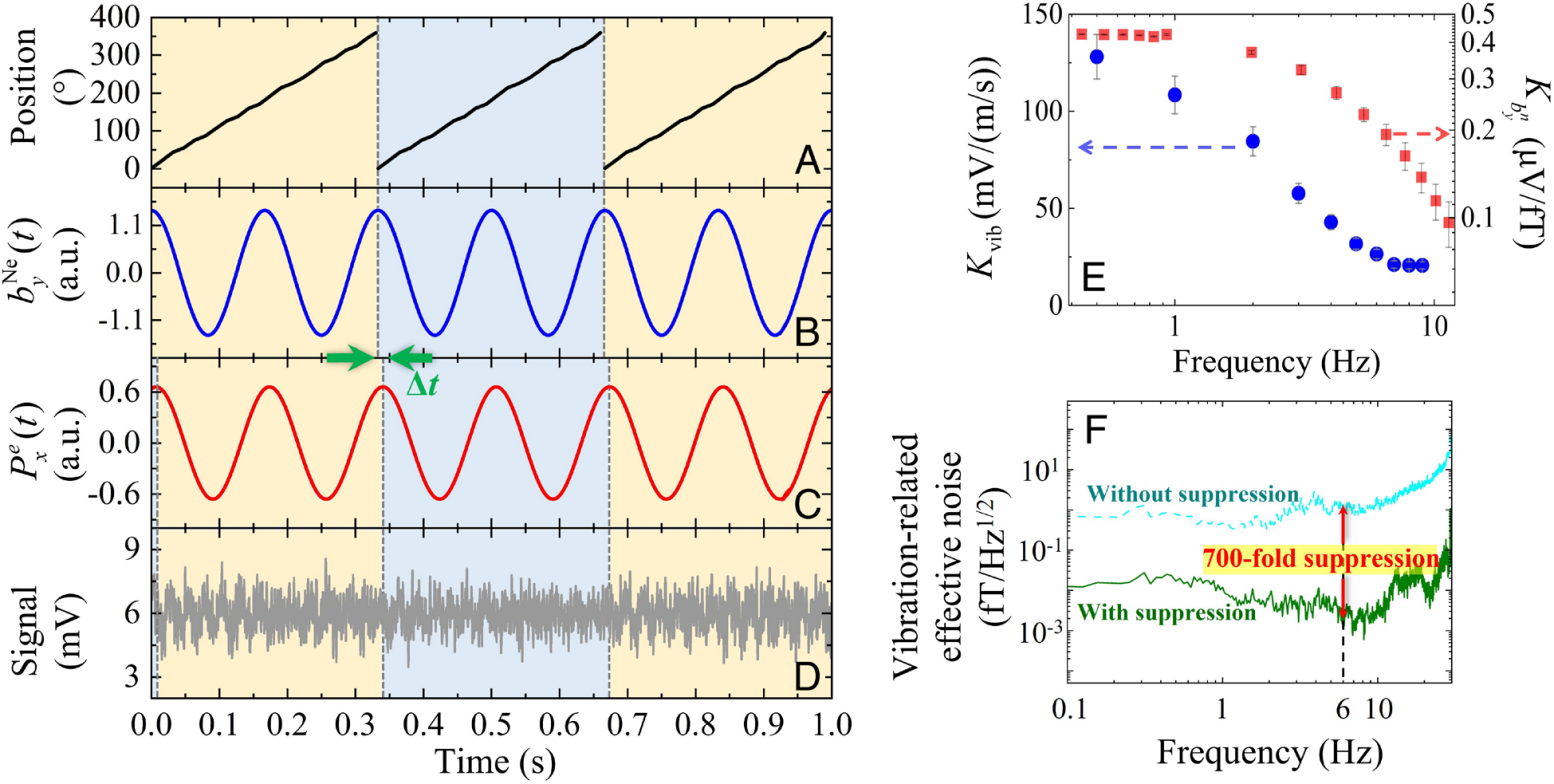
Data acquisition and simulation process. (*A*) The optical encoder measures the rotation angle (0° to 360°) in real-time to obtain the position of Lead 1. An angle of 0º indicates that Lead 1 is at the lowest point. (*B*) As the motor rotates, the pseudomagnetic field $b_{y}^{\mathrm{Ne}}$ of double frequency induced by the two lead blocks is simulated. (*C*) The simulated response of the comagnetometer to $b_{y}^{\mathrm{Ne}}$ displays a delay Δt, which corresponds to the phase relative to the $b_{y}^{\mathrm{Ne}}$ input. (*D*) The measured response of the comagnetometer. Vibration noise in the experiment is observed and multistage vibration suppression is conducted. (*E*) Calibrated transduction coefficients for vibration and exotic field $b_{y}^{\mathrm{Ne}}$ to comagnetometer response. Blue circles denote the vibration-to-response transfer coefficient *K*_vib_ with multistage vibration suppression (left vertical axis, calibration procedure detailed in Ref. 25). Red squares represent the $b_{y}^{\mathrm{Ne}}$-to-response transfer coefficient $K_{b_{y}^{n}}$ (right vertical axis), and $K_{b_{y}^{n}}$ is calibrated with classical magnetic fields. (*F*) Comparison of vibration-related effective noise with (cyan line) and without (olive line) multistage vibration suppression. Multistage vibration suppression comprises the first-level vibration isolation foundation and the second-level vibration suppression. At the pseudomagnetic field modulation frequency, the vibration-related effective noise of the HSR-based comagnetometer without multistage vibration suppression is approximately 1.33 fT/Hz^1/2^@6 Hz, while the noise is approximately 1.83 aT/Hz^1/2^ at the same frequency with multistage vibration suppression. The multistage vibration suppression achieves over 700 times reduction in vibration-related noise. Vibration-related effective noise is obtained by converting the measured vibration noise (using a seismometer) via the coefficients presented in (*E*).

The experimental setup is depicted in Fig. 1*B*. Two cubic lead blocks, each with a side length of 10.00 cm, are mounted on the axis of a high-power servo motor. The lead blocks have a high mass density of 11.3 g/cm^3^, resulting in a correspondingly high nucleon density (6.8 × 10^24^ /cm^3^) (13), which makes them an ideal choice of mass source (22–24). The servomotor rotates the two lead blocks at approximately 3 Hz, corresponding to the exotic force modulation at 6 Hz due to symmetry. The centers of the lead blocks rotate in a circle with a radius of 50.0 cm, with the bottom plane of the blocks aligned with the atomic vapor at the lowest point of rotation. An optical encoder is used to monitor the rotational angle in real time with a precision of ±4.9 μrad, and one set of data is shown in Fig. 2*A*.

The K–Rb–^21^Ne comagnetometer used in this experiment is similar to that described in Refs. 20, 26. A 12-mm diameter spherical cell, containing 2280 torr of ^21^Ne, 70 torr of N_2_, and K/Rb vapor (density ratio ∼1:100 at 195 °C), is enclosed within a five-layer magnetic shield *μ*-metal and Mn-Zn ferrite. Hybrid optical pumping along the *z* axis enhances the polarization uniformity of the alkali spin and the hyperpolarization efficiency of the noble gas spins: A circularly polarized resonant laser directly polarizes the K atoms, while spin-exchange collisions transfer polarization to Rb atoms and ^21^Ne nuclei. The exotic force can couple to the electron, proton, and neutron spins in the alkali atoms. Here, we take the ^21^Ne nuclei as an example. The precession of ^21^Ne nuclei, induced by the parity-violating pseudomagnetic field $b_{y}^{\mathrm{Ne}}$, generates a real magnetic field that can be detected by Rb atoms. The resulting dynamics of the Rb atoms are measured via the optical rotation of off-resonant polarized light propagating along the *x*-direction. The exotic field $b_{y}^{\mathrm{Ne}}$ generated by the source mass can be inferred from the measured optical rotation signal with a conversion factor $K_{b_{y}^{n}}$, which is detailed in Eq. **2**.

### HSR Regime.

In long-term precision measurements, the drift of system parameters and variations in environmental conditions can significantly affect the accuracy and stability of measurement results. This necessitates a careful balance between high sensitivity and long-term stability in the measurement of weak pseudomagnetic fields. Operating the coupled atomic ensemble in the resonantly-coupled HSR regime is particularly suitable for this purpose. By applying a static magnetic field *B_z_* along the *z*-axis (the spin-polarization axis) with a magnitude equals to the magnitude of the effective magnetic field from the Fermi-contact interactions between the Rb and ^21^Ne atoms (27), the Larmor precession of the ^21^Ne spins and the Rb atoms becomes strongly coupled. In this scenario, the relaxation of noble-gas spins is influenced by alkali spins, resulting in a broader bandwidth of nuclear spins, and the regime exhibits superior stability across a broad frequency range (details can be found in *Materials and Methods*). The response of the comagnetometer to the oscillating exotic field coupled to noble-gas spins, represented as $b_{y}^{\mathrm{Ne}}\left(t\right)=b_{y0}^{\mathrm{Ne}}\cos\left(\omega t\right)$, can be expressed as:

[2] $$P_{x}^{e}(t)=K_{b_{y}^{n}}b_{y0}^{\mathrm{Ne}}\cos(\omega t-\phi_{b_{y}^{\mathrm{Ne}}}),$$

where $K_{b_{y}^{n}}$ relates the pseudomagnetic field with the spin polarization along *x*-axis. This factor depends on the longitudinal spin polarizations $P_{z}^{e/n}$, the transverse relaxation rates of both spins and the effective magnetic fields from Fermi-contact interactions (see details in (27)), and $\phi_{b_{y}^{\mathrm{Ne}}}$ presents the phase shift of the optical rotation signal due to $b_{y}^{\mathrm{Ne}}$. In experimental setups, classical magnetic fields are typically employed to calibrate the response factor of the atoms to the exotic field $K_{b_{y}^{n}}$. This methodology is commonly utilized in experiments seeking new physics beyond the Standard Model (20, 28).

The HSR regime of the comagnetometer demonstrates a bandwidth up to 25 Hz, shown in Fig. 5*A*, which tends to cover multiple harmonic components of $b_{y}^{\mathrm{Ne}}$. By changing the rotation frequency of the lead blocks, exotic signals are in the frequency region where the noise performance of the HSR comagnetometer is optimal. Utilizing the methodology outlined in (29), we simulated the pseudomagnetic signal $b_{y}^{\mathrm{Ne}}$, with the primary input parameters for the simulation detailed in Table 1. The simulated response is illustrated in Fig. 2*B*. The modulated exotic field generated by the source mass can be detected by the comagnetometer as $b_{y}^{\mathrm{Ne}}=\zeta^{n,p}/\mu_{\mathrm{Ne}}\int \rho_{N}V_{\mathrm{PV}}dV$, where $\zeta^{n}=0.58$ and $\zeta^{p}=0.04$ are the fraction factors for neutron and proton spin polarization in the ^21^Ne nucleus (29, 30), while $\mu_{\mathrm{Ne}}$ stands for the magnetic moment of the ^21^Ne nucleus, and $\rho_{N}$ is the nucleon density which is the average number of neutron and protons in the mass source. Fig. 2*C* presents the simulated $P_{x}^{e}\left(t\right)$, derived from Eq. **2**, employing the calibrated parameters of the K–Rb–^21^Ne ensemble in *Materials and Methods*. We further investigate the phase shifts between the HSR response and the exotic field $b_{y}^{\mathrm{Ne}}$. At a modulation frequency of 6 Hz (corresponding to rotation frequency of 3 Hz), the phase shift $\phi_{b_{y}^{\mathrm{Ne}}}={10.1\pm 5.6}^{\circ }$ corresponds to a time delay Δt=4.7±2.6 ms, which aligns with the respective signal segments illustrated in Fig. 2 *B* and *C*. Fig. 2*D* displays the corresponding response of the comagnetometer, where fluctuations in the experimental data are attributed to resonance vibrations of the mechanical structure of the setup. The vibration-related noise, a primary source of uncertainty in the experiment, is mitigated by an overall factor of 700 (shown in Fig. 2*F*) through a multistage isolation approach. Please note that the vibration-related noise represents the mechanical noise, which encompasses ground vibrations, air-convection noise, and airborne acoustic noise.

**Table 1.** Summary of calibrated parameters and systematic errors

Parameter	Value	$\Delta g_{A}^{n}g_{V}^{N}$ (×10^−39^)
Mass of lead *M* (kg)	12.00 ± 0.01	<0.01
Position of *X* (cm)	52.5 ± 5.5	0.28
Position of *Y* (cm)	0.0 ± 1.0	<0.05
Position of *Z* (cm)	0.0 ± 1.0	<0.05
Modulation frequency $f_{m}$ (Hz)	6.00 ± 0.14	0.06
Ring radius *R* (cm)	50.0 ± 1.0	0.06
Calibrated $K_{b_{y}^{n}}$ (μV/fT)	0.193 ± 0.016	0.77
Phase uncertainty $\phi_{b_{y}^{\mathrm{Ne}}}$ (°)	10.1 ± 5.6	+1.4
		−2.3
Vibration-equivalent magnetic noise (aT/Hz^1/2^)	<1.83	<5.4
Final $g_{A}^{n}g_{V}^{N}$ (×10^−39^)	4.76	5.88 (syst)
(*λ* = 5 m)		12.34 (stat)

The corrections to $g_{A}^{n}g_{V}^{N}$ for λ = 5m are listed.

### New Constraints on Parity-Odd Interactions.

**Fig. 3.**
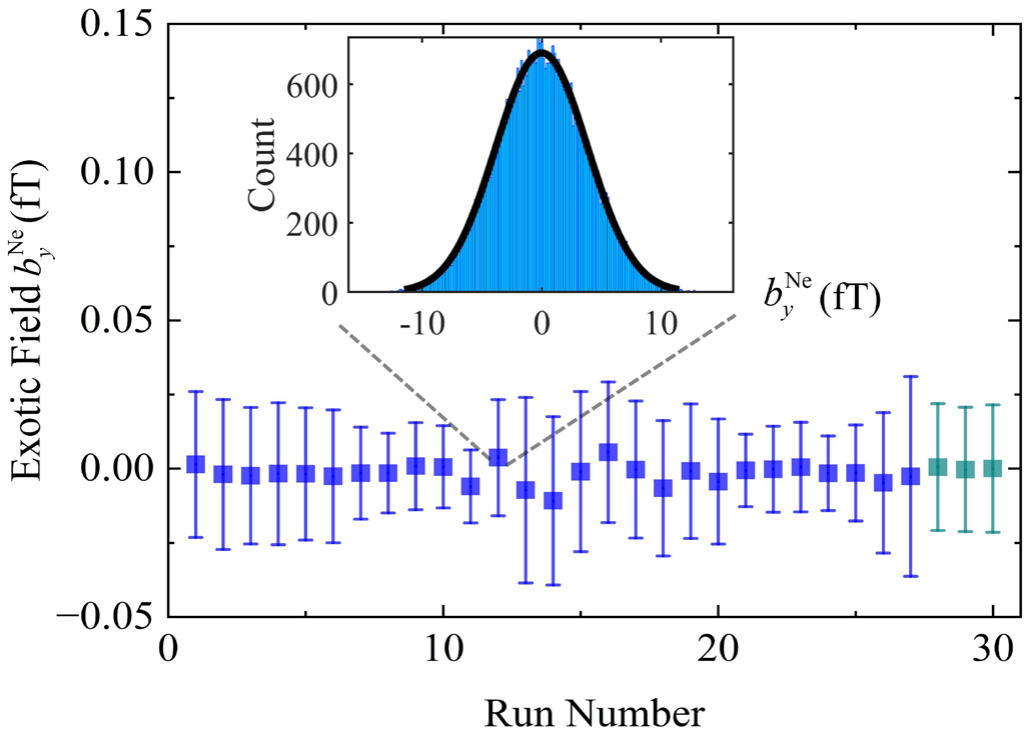
Experimental results of the exotic field $b_{y}^{\mathrm{Ne}}$. Blue points denote measurements with the mass source rotating clockwise for 108 h; cyan points represent a control experiment with counter-clockwise rotation for 12 h. Each point represents an average of about one 4-h dataset. The error bars represent the statistical properties of the tested results within one 4-h dataset. The distribution of $b_{y}^{\mathrm{Ne}}$ for one data set is shown in the inset, with the black curve being a Gaussian fit with reduced $\chi^{2}=1.16$. The exotic field $b_{y}^{\mathrm{Ne}}$ is measured to be (1.615 ± 4.183_stat_) aT.

The total duration for collecting the pseudomagnetic field is 108 h with the mass source rotating clockwise, during which the measurements are conducted in time series of 4 h intervals to ensure calibration stability and experimental convenience (29). Additionally, a 12-h control experiment with counterclockwise rotation is performed. The amplitude of the dominant harmonic components of the pseudomagnetic field is analyzed with a weighting method (please refer to 31–33) to mitigate potential slow drifts within the system, and weighted processing is performed according to the Fourier coefficients $c_{k}$ of each harmonic component (see in *Materials and Methods*). The measured $b_{y}^{\mathrm{Ne}}$ is found to be (1.61 ± 4.18_stat_ ± 1.99_syst_) aT, as depicted in Fig. 3. The results account for various system uncertainties presented in Table 1, including statistical uncertainty and systematical errors related to source mass, position, rotation radius, modulation frequency, calibration factor, and signal phase. The primary systematic error arises from vibrations, especially the acoustic coupling between source-mass rotation and output signals. Measurements using a seismometer indicate that the mass source system induced peak ground vibration is approximately 3.9 × 10^−7^ m/s/Hz^1/2^. To mitigate this major systematic error in measurements, we install the HSR comagnetometer on an isolated foundation to reduce the ground vibration to be 1.34 × 10^−8^ m/s/Hz^1/2^ and design a specialized split vacuum chamber to further suppress the vibration-related noise (25, 34). Multistage isolation effectively suppresses vibration-related noise by over a factor of 700. With the calibrated coefficients in Fig. 2*E*, a vibration-related magnetic field of 1.83 aT is estimated, ultimately constraining $\Delta g_{A}^{n}g_{V}^{N}<5.4\times 10^{-39}$ as listed in Table 1.

**Fig. 4.**
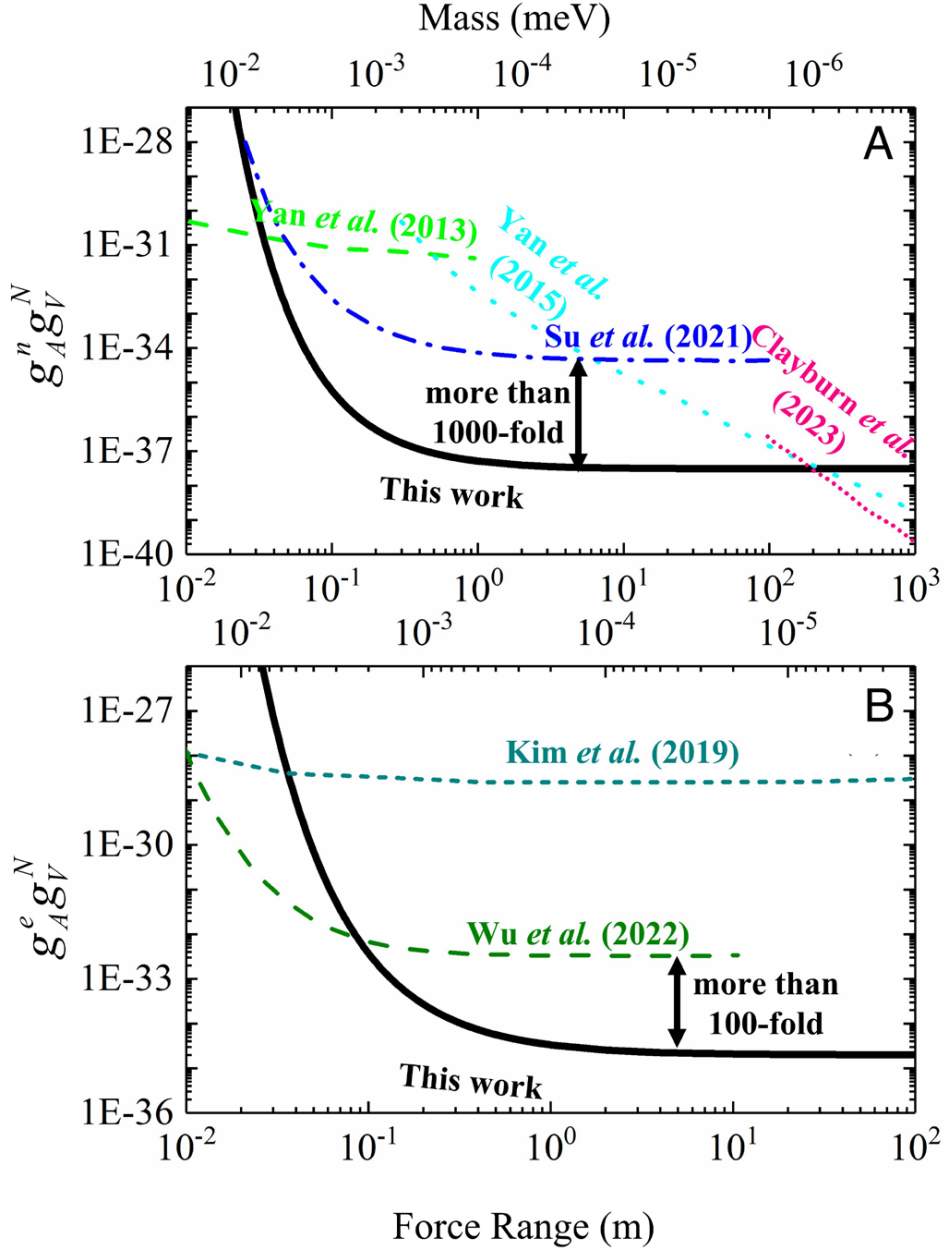
The experimental limits on coupling coefficients. (*A*) Constraints on the coupling constants $g_{A}^{n}g_{V}^{N}$ (95% confidence level) as a function of interaction force range. The black solid line is the current constraints. The green dashed line, “Yan et al. (2013)” is from Ref. 37 that search for parity violation in neutron spin rotation in liquid ^4^He; the cyan dotted line, “Yan et al. (2015)” is from Ref. 21 that considers the Earth as an unpolarized source; and the “Clayburn et al. (2023)” from Ref. 18, the pink dotted line, uses Earth to search for long-range spin-velocity interactions; the blue dashed-dotted line, “Su et al. (2021)” is from Ref. 17 that uses a spin-based amplifier. (*B*) Constraints on the coupling constants $g_{A}^{e}g_{V}^{N}$ (95% confidence level) as a function of interaction range. The black solid line is the current constraints. The dark-cyan dashed line, “Kim et al. (2019)” is from Ref. 36 that take the fraction of electron spin into account; the olive dashed line, “Wu et al. (2022)” is from Ref. (35) that uses two BGO masses and an atomic magnetometer array, where we make a modification that considers the fraction of electron spin as well.

Fig. 4 illustrates the new limits on the dimensionless coupling coefficient established by the experiment. Considering the contribution of neutron spins in ^21^Ne, we established the strongest constraints on the interaction between neutrons and nucleons, as depicted by the solid black line in Fig. 4*A*. At a force range of λ=5 m, the coupling constant is $g_{A}^{n}g_{V}^{N}$ = (4.76 ± 12.34_stat_ ± 5.88_syst_) × 10^−39^, signifying a three-orders-of-magnitude improvement over previous constraints (17). The 95% confidence level of |$g_{A}^{n}g_{V}^{N}$| ≤ 2.83 × 10^−38^ is determined via Gaussian-based Monte Carlo simulations, where random samples generated from normal distributions defined by the measured parameters were analyzed to establish the coverage interval containing 95% of the probability mass. Our findings set the currently most stringent limits on parity-odd interactions within a force range of 0.03 to 400 m. Additionally, the exotic force coupling to electron spins in the comagnetometer can be deduced with the same method as the ^21^Ne. Please note that when we consider the coupling to a specific fermion in the sensor, for example, electron spins or protons, we assume that couplings to all other fermions are zero. We need to replace the magnetic moment of ^21^Ne to Rb, and use the response curve of the Rb atoms to the exotic field. For Rb atoms with 50% polarization, the proton’s fraction of spin is approximately 0.29, while the electron’s is about 0.13 (33). We present the result for *e−N* in Fig. 4*B*. The corresponding result for *p−N* can be obtained by rescaling the line of *e−N* according to the ratio of their fraction of spin. We obtained $b_{y}^{e}$ =(0.070±0.181_stat_±0.171_syst_) aT, corresponding to a coupling constant $g_{A}^{e}g_{V}^{N}$ = (2.50 ± 6.69_stat_ ± 6.11_syst_) × 10^−36^ at λ=5 m, establishing a limit at the 95% confidence level of |$g_{A}^{e}g_{V}^{N}$| ≤ 1.84 × 10^−35^, representing an enhancement of more than two orders of magnitude over the previous limit established in Ref. 35. Please note that the Ref. 35 and Ref. 36 didn’t take the fraction of spin into account, and we rescaled their result in the plot with fraction of spin accordingly.

Apart from the studies presented in Fig. 4, there have also been investigations of exotic forces at much shorter ranges. For example, parity-violation experiments have placed stringent limits on electron–neutron couplings over force ranges from sub-femtometer to millimeter scales (15). At the opposite extreme, other works have used the Sun and Moon as sources to search for forces with ranges exceeding 10^10^ km (19). Our measurements establish the strongest constraints on axial-vector couplings in the range of 0.03 m to 400 m. This improvement significantly narrows the parameter space of the exotic force, excluding regions inaccessible to short-range precision spectroscopy as well as constraints from astrophysical observations at larger interaction ranges.

## Discussion

**Fig. 5.**
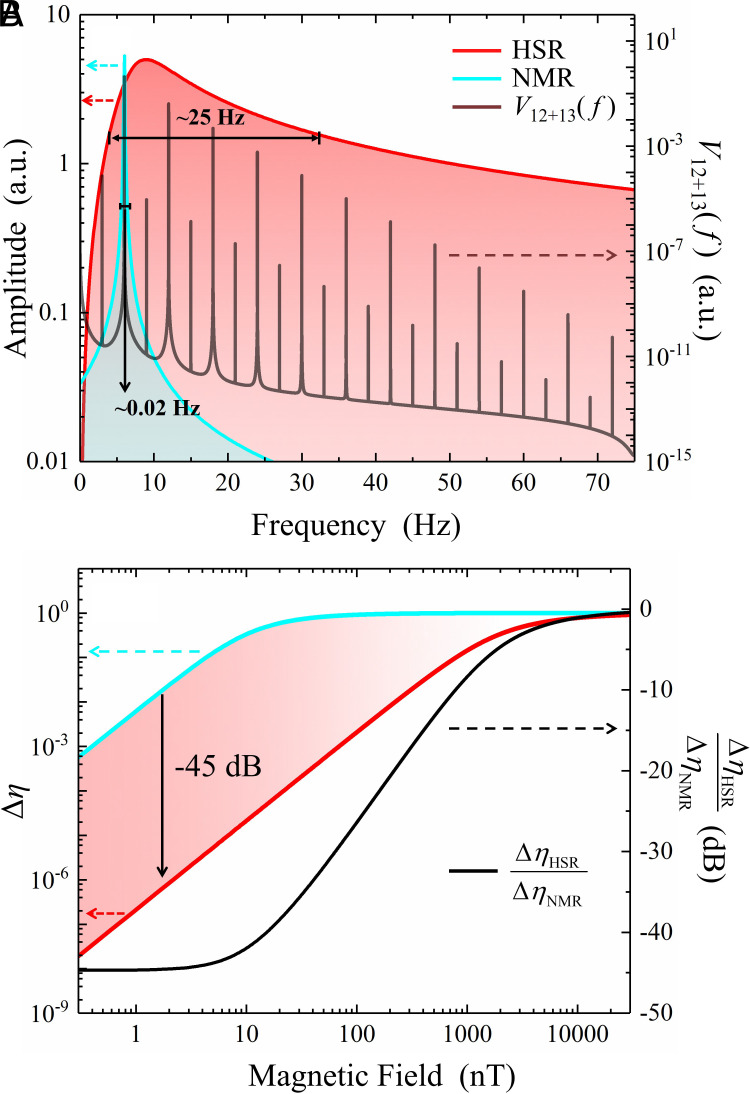
Effective bandwidth and disturbance rejection of K–Rb–^21^Ne comagnetometers operating in different regimes. (*A*) Comparison of bandwidths in HSR (red line) and NMR (cyan line) regimes. The bandwidth of HSR is more than three orders of magnitude greater than that of NMR, which can effectively cover multiple harmonic components of the potential *V*_12+13_ (gray line). The red and cyan lines are plotted against the left vertical axis, while the gray line uses the right vertical axis. (*B*) Comparison of disturbance rejection in the HSR and NMR regimes. The relative change in output amplitude of the system under magnetic fluctuations can be quantitatively analyzed using the rejection ratio Δη in Eq. **3**. Δη close to zero indicates strong disturbance rejection. The colored gradient areas illustrate the advantages of HSR over NMR in terms of disturbance rejection. The black curve, which is plotted against the right vertical axis, represents $\Delta \eta_{\mathrm{HSR}}/\Delta \eta_{\mathrm{NMR}}$. Within the fluctuation range of 0.3 to 10 nT [for ^3^He (22) ranges from 0.03 to 1 nT, while 0.09 to 3 nT for ^129^Xe (17)], the disturbance rejection of HSR is enhanced by 45 dB compared to NMR.

In contrast to previous experiments relying on self-compensation (SC) (22) or nuclear magnetic resonance (NMR) (17) modes for exotic field detection, the HSR regime enhances measurement stability by broadening the sensor bandwidth while retaining the exceptional sensitivity of SERF comagnetometers. Demonstrated results show a 45 dB improvement in disturbance rejection (see Fig. 5*B* for details). Unlike the SC mode that suppresses low-frequency magnetic noise by balancing external and effective magnetic fields ($B_{z}=-B_{z}^{e}-B_{z}^{n}$), the HSR regime operates under the condition $B_{z}\approx -B_{z}^{n}$ (20), enabling synchronized dynamics between alkali and noble-gas spin ensembles. The magnetic suppression factor, defined as the ratio of responses to a classic magnetic field $B_{y}$ and a pseudomagnetic field $b_{y}^{\mathrm{Ne}}$ (14), reveals five-fold suppression for slowly varying magnetic fields below 40 mHz. This suppression capability is indispensable for long-term stability, as it reduces the parameter drifts induced by environmental perturbations (e.g., temperature variations and light-intensity fluctuations) over extended measurement durations. At the same time, the HSR regime inherits the signal amplification advantage of NMR, where spin-ensemble coupling induces an approximately 100-fold enhancement of the effective magnetic field sensed by alkali spins within the HSR frequency range. By simultaneously extending the bandwidth and improving the signal-to-noise ratio (SNR), the HSR regime offers a robust technique for precision measurements.

A major challenge in this experiment arises from vibration-related noise induced by mass motion. The multistage isolation, integrating the vibration isolation foundation with split vacuum system, totally achieves 700-fold suppression of vibration noise, sheding light on quantum technologies requiring subpicometer stability such as gravitational wave interferometry (38). Future research will optimize pseudomagnetic field detection through enhanced spin-coherence materials and multilayer magnetic shielding architectures. Precision metrology incorporating quantum control protocols and machine learning-assisted noise suppression (39) will minimize systematic uncertainties. Implementation of nonclassical spin states (40) promises to surpass standard quantum limits, thereby extending applications in fundamental interaction studies.

In this work, we demonstrate a resonantly-coupled HSR regime in atomic ensembles to probe parity-violating interactions, improving constraints on the axial–vector coupling $g_{A}g_{V}$ by three orders of magnitude. This advance establishes the HSR method as a powerful tool for precision tests of fundamental symmetries. Future refinements may extend its sensitivity to beyond-Standard-Model physics and other exotic spin-dependent interactions. The presented experiment continues the seven-decade tradition of atomic parity violation studies that started with the visionary proposal of Ya.B. Zel’dovich (41) and is augmented today with the searches for long-range exotic parity-violating forces with ultrasensitive atomic magnetometers, as presented here.

## Materials and Methods

### Stability Enhancement.

In the resonantly-coupled HSR regime, the comagnetometer maintains its ultrahigh sensitivity while achieving a bandwidth on the order of tens of hertz. Compared to NMR magnetometers, the achieved high bandwidth can cover the multiple harmonic components of the pseudomagnetic field, as illustrated in Fig. 5*A*.

Comagnetometers operating in the HSR regime demonstrate superior disturbance rejection capabilities across a wide frequency range. To quantitatively assess the disturbance rejection performance of the magnetometer, we utilize the rejection ratio, a metric commonly employed in the field of control systems. The rejection ratio is defined as the ratio of the disturbance signal relative to that of the reference input signal. It can be mathematically expressed as

[3] $$\Delta \eta =\left|\frac{A(f_{0})-A(f_{0}+\Delta f)}{A(f_{0})}\right|,$$

where $A(f_{0})$ represents the response of the magnetometer to the resonant frequency $f_{0}$ and Δf indicates the fluctuations in $f_{0}$ caused by variations in experimental parameters such as atomic density, magnetic field, and light intensity. When the K–Rb–^21^Ne magnetometer operates in the HSR regime, the response $A_{\mathrm{HSR}}\propto fQ/\sqrt{{\left(R_{2}^{e}/f\right)}^{2}+{\left({\left(fQ\right)}^{2}-\gamma_{e}\gamma_{n}B_{z}^{e}B_{z}^{n}\right)}^{2}}$ (20, 27), where $\gamma_{e/n}$ denote the gyromagnetic ratios for electrons and nuclei, respectively, while $B_{z}^{e/n}$ represent the effective magnetic fields on electronic and nuclear spins arising from Fermi-contact interactions between them, and Q is the slowing-down factor for alkali atoms (14, 26). In contrast, for the NMR regime, $A_{\mathrm{NMR}}\propto 1/\sqrt{{\left(R_{2}^{n}\right)}^{2}+{\left(f-\gamma_{n}B_{z}^{\mathrm{eff}}\right)}^{2}}$ (17, 42), where $R_{2}^{e/n}$ are the transverse relaxation rates of the alkali spin and the noble-gas spin, and $B_{z}^{\mathrm{eff}}$ denotes the total effective magnetic field detected by the ^21^Ne nuclei. The rejection ratios for the two regimes are illustrated in Fig. 5*B* with calibrated parameters of the K–Rb–^21^Ne ensemble: $R_{2}^{n}$ ≈ 0.005 s^−1^, $R_{2}^{e}$≈ 3900 s^−1^, Q ≈ 7.6, $B_{z}^{e}$ ≈ 83 nT, $B_{z}^{n}$ ≈ 468 nT, and $B_{z}^{\mathrm{eff}}$ ≈ 1.8 pT.

As shown in Fig. 5*B*, the disturbance rejection of both regimes degrades significantly when magnetic deviations exceed the response bandwidths. The HSR regime, with a bandwidth of tens of hertz (∼10 μT), outperforms the NMR regime, which operates at millihertz bandwidths (∼10 nT). The broader bandwidth in HSR enables effective suppression of frequency fluctuations caused by the environmental parameters such as the residual magnetic fields and nonconstant temperature, enhancing pseudomagnetic field measurement stability. For weak pseudomagnetic fields, even minor frequency drifts can severely degrade fidelity, potentially rendering prolonged measurements unreliable. HSR not only improves disturbance rejection but also maintains signal integrity over extended durations. Within a 0.3 to 10 nT fluctuation range, HSR achieves a 45 dB improvement over NMR, highlighting its potential for high-precision applications requiring robust stability against environmental noise.

### Data Processing.

The data processing methodology is consistent with the approach delineated in Ref. 35, 43. The equivalent pseudomagnetic field comprises multiple harmonic components with a fundamental frequency denoted by $f_{m}$, as illustrated in Fig. 5*A*, and the mathematical representation of the measured signal can be expressed as $b\left(t\right)=g_{A}g_{V}\sum_{k}\left[c_{k}\cos\left(2\pi kf_{m}t-\varphi \right)\right]+n\left(t\right)$, where $c_{k}$ denotes the Fourier coefficients of the various harmonic components and can be obtained through numerical integration. The term φ corresponds to the initial phase factor of the system, derived from the phase signal of the encoder, while n(t) accounts for noise in the measurement. Within the bandwidth, the interaction coupling constant $g_{A}g_{V}$ can ultimately be determined as $g_{A}g_{V}=\sum_{k}\left(c_{k}^{2}g_{A}g_{V}{|}_{k}\right)/\sum_{k}\left(c_{k}^{2}\right)$ (similar in 43). Here, the *k*-th harmonic interaction coupling constant is defined by

[4] $${\left.g_{A}g_{V}\right|}_{k}=\frac{2f_{m}}{c_{k}M}\times \int_{0}^{M/f_{m}}\cos\left(2\pi kf_{m}t-\varphi \right)b\left(t\right)dt$$

with $M/f_{m}$ being the total observation time encompassing M cycles. The numerical simulation in Fig. 5*A* presents normalized $c_{1}:c_{2}:c_{3}\approx 1.00:0.09:0.01$, with other harmonic components being neglected. The adoption of multiharmonic measurements in the HSR regime, as opposed to relying solely on the fundamental frequency or a single harmonic component, shows a potential to differentiate signal characteristics, thereby enhancing the signal-to-noise ratio (SNR) (35).

Both clockwise rotation data (108 h) and counterclockwise control data (12 h) are segmented into 4-hour datasets. The final pseudomagnetic field is derived by statistically combining the expectation values from clockwise runs with the sign-inverted counterclockwise expectations, weighted by their respective uncertainties.

### Experimental Details.

**Fig. 6.**
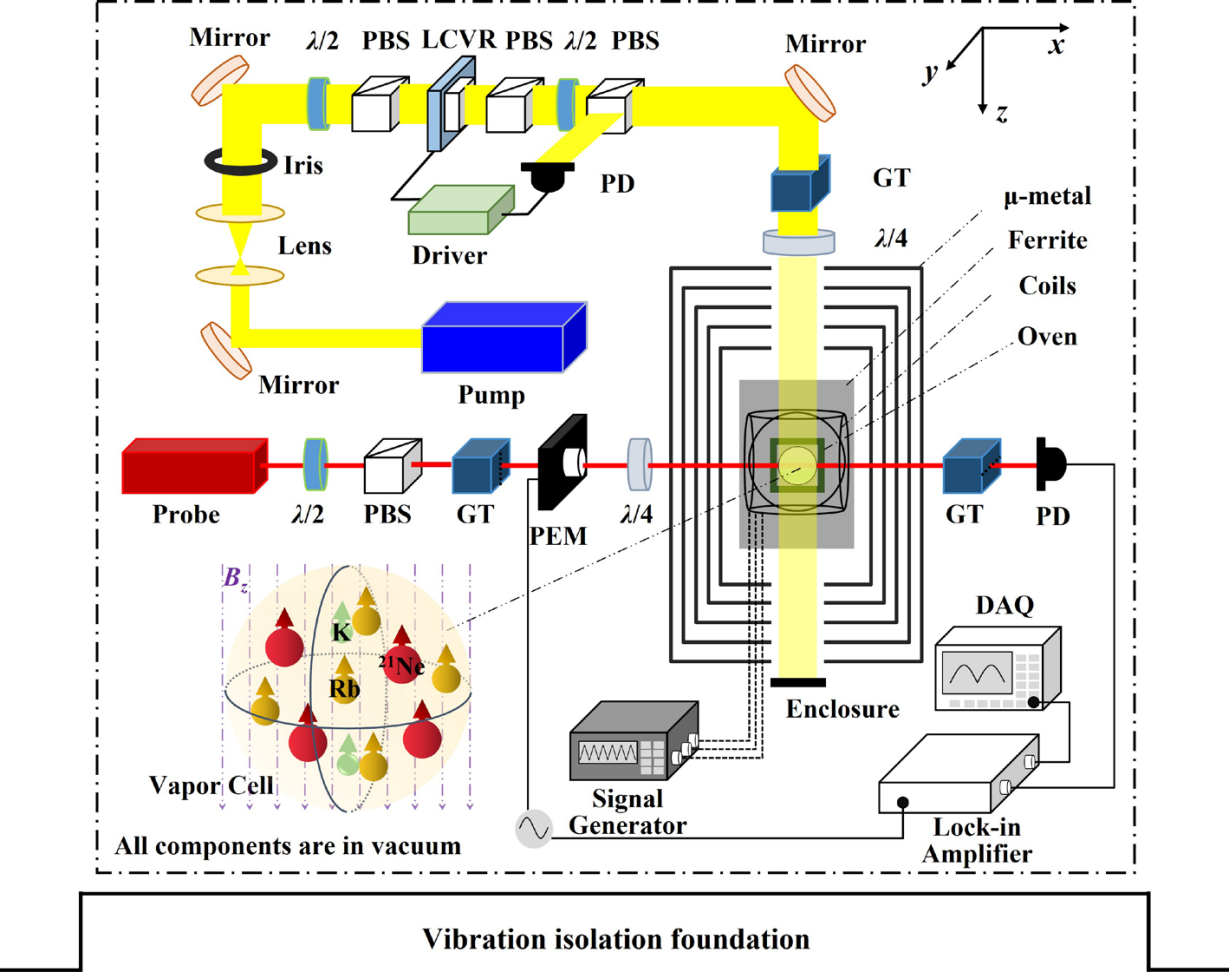
Schematic of experimental details. The pump beam is collimated using two focal-length-matched lenses and shaped to a 14-mm diameter by an aperture to fully illuminate the vapor cell. The probe beam, propagating along the *x*-axis, passes sequentially through a Glan–Taylor polarizer (extinction ratio >10^5^), a Photoelastic modulator oriented at 45°, and a quarter-wave plate oriented at 0° before entering the cell for Faraday rotation measurement. After the cell, the probe beam passes through an analyzer orthogonal to the polarizer and is detected by a photodiode. The resulting photocurrent is demodulated by a lock-in amplifier to extract the fundamental-frequency component. The demodulated signal amplitude is proportional to the Faraday rotation angle, enabling quantitative measurement of the pseudomagnetic field signal. *λ*/2: Half-wave plate. *λ*/4: Quarter-wave plate. PBS: Polarizing beam splitter. GT: Glan-Taylor polarizer. PEM: Photoelastic modulator. LCVR: Liquid crystal variable retarder. PD: Photodiode detector. DAQ: Data acquisition system.

The experimental setup is detailed in Fig. 6. A vapor cell containing K–Rb–^21^Ne–N_2_ mixtures is positioned at the center of the multilayer magnetic shield. The cell is heated using a non-magnetic electric heater. Thermal insulation, provided by surrounding the cell with the Polymethacrylimide material, ensures temperature uniformity. Three pairs of orthogonal Helmholtz coils, arranged around the cell, generate a stable, homogeneous magnetic field environment. A static bias field of *B_z_* = 468 nT (equaling to $B_{z}^{n}$) is applied to operate the K–Rb–^21^Ne spin ensemble in the HSR regime.

The wavelength of pump light is locked to the K D1 resonance line at 770.108 nm using saturation absorption spectroscopy. The intensity is dynamically controlled and stabilized using a liquid crystal variable retarder (LCVR). The pump beam is converted to circular polarization by a quarter-wave plate. After optically pumping the K atoms, polarization is transferred to the Rb atoms and subsequently to the ^21^Ne nuclei via spin-exchange collisions, resulting in Rb polarization and ^21^Ne hyperpolarization.

The linearly polarized probe light, centered at 795.5 nm (red-detuned by 237.2 GHz from the Rb D1 line), measures Rb spin precession induced by the pseudomagnetic field via the Faraday rotation effect. The probe noise floor reached 0.1 fT/Hz^1/2^, approaching the theoretical photon shot noise limit (44).

The entire comagnetometer is mounted on a vibration isolation foundation. Upon activation of the split vacuum system, the chamber achieves pressures below 50 mBar, effectively isolating the system from vibration-related noise induced by the rotating masses.

## Data Availability

All study data are included in the main text.
